# Breakfast skipping is related to inadequacy of vitamin and mineral intakes among Japanese female junior high school students: a cross-sectional study

**DOI:** 10.1017/jns.2019.44

**Published:** 2020-02-10

**Authors:** Mai Matsumoto, Yoichi Hatamoto, Azusa Sakamoto, Ayumi Masumoto, Shinji Ikemoto

**Affiliations:** 1 Department of Nutritional Epidemiology and Shokuiku, National Institutes of Biomedical Innovation, Health, and Nutrition, Tokyo, Japan; 2 Department of Nutrition and Metabolism, National Institutes of Biomedical Innovation, Health, and Nutrition, Tokyo, Japan; 3 HANA College of Nutrition, Tokyo, Japan; 4 Saitama City, Saitama, Japan; 5 Department of Human Nutrition, Seitoku University, Chiba, Japan

**Keywords:** Breakfast skipping, Adolescence, Nutritional adequacy, Habitual nutrient intakes, BDHQ15y, brief self-administered diet history questionnaire for Japanese children and adolescents, DG, dietary goal to prevent lifestyle-related diseases, DRI, dietary reference intake, EAR, estimated average requirement

## Abstract

Breakfast skipping is a public health issue which affects nutrient intake among adolescents worldwide. However, there have been few reports comparing intake and reference values to assess the deficiency of nutrient intake between breakfast consumers and skippers. Therefore, the present study aimed to examine the relationship between breakfast skipping and adequacy of total habitual nutrient intake among junior high school female students. The participants were 516 Japanese female junior high school students. Dietary habits during the preceding month were assessed using a brief self-administered diet history questionnaire. Inadequacy of each nutrient intake was assessed by the cut-point method, based on the estimated average requirement for fourteen nutrients and on dietary goal values for five nutrients. The overall nutritional inadequacy in participants was assessed by the number of consumed nutrients which did not meet the requirements as per the dietary reference intakes for Japanese, 2015 version. The participants were classified into two groups according to the frequency of breakfast eating: breakfast consumers (seven times/week) and breakfast skippers (0–6 times/week). Adequacy of vitamin A, vitamin B_1_, vitamin B_2_, vitamin C, Ca, Fe, Zn and K was higher among breakfast consumers than among skippers. Breakfast consumers had more intakes of fruits, vegetables and dairy products. Our findings suggest that breakfast skipping was related to deficiencies in vitamin and mineral intakes, and to an unfavourable dietary pattern, among Japanese female junior high school students.

Adolescence is a crucial period for physical and mental development, and the diet and eating behaviours which develop during these years tend to persist throughout the individual's life^(1–3)^. The eating habits and behaviours established during adolescence may be difficult to change later in life^(4)^. Establishing healthy food consumption behaviours leads to nutritional adequacy, and eating behaviours influence morbidity and mortality in adolescence and in adulthood^(5)^.

Breakfast skipping is reported to be one of the bad eating habits, and affects an individual's health status. For example, breakfast skipping was reported to be associated with weight gain^(6,7)^ and increase of BMI^(8,9)^. Moreover, breakfast skipping was also reported to be associated with a higher prevalence of obesity^(7)^, hypertension^(10,11)^, hyperlipidaemia^(11,12)^, insulin insensitivity and type 2 diabetes^(7)^ and CVD^(11,13)^. Such reports might partly explain findings from previous cross-sectional and intervention studies which show that eating breakfast may be a predictor of healthy eating behaviour, and implies a high intake of dietary fibre, thiamine or folate, and a low intake of fat^(14–16)^. A systematic review of studies about breakfast skipping reported that one of the main factors for breakfast skipping was habituation^(17)^. The rate of breakfast skipping among Japanese adolescents has not decreased over the past 10 years and is slightly less than that of adults^(18,19)^. Thus, it is highly likely that breakfast skipping continues as a habit from adolescence to adulthood. Similarly, breakfast skipping has been reported as a public health issue worldwide, including in the USA^(20)^ and the UK^(21)^. Therefore, breakfast skipping in adolescence is accepted as one of the important public nutritional problems in the world.

There are a few reports about the association between breakfast consumption and the adequacy of nutrient intake among adolescents. For example, a UK study has reported that the intakes of folate, vitamin C, Ca and iodine for breakfast consumers were higher than those of breakfast skippers among children and adolescents aged 4–18 years; in addition, the proportion of breakfast consumers aged 4–18 years having adequacy of folate, vitamin C, Ca, Fe and iodine was higher among breakfast consumers^(21)^. However, only specific nutrients were selected for comparison with reference values in this previous study. Additionally, a Belgium study reported that the consumers of a good-quality breakfast rich in cereals, dairy products and fruits/vegetables had higher intakes of protein, Fe, Ca, Mg, vitamin B_1_, vitamin B_2_ and vitamin C than did those who consumed breakfast of a lower quality such as only drinking a beverage with energy or those of less than 1674 kJ/meal (400 kcal/meal) among adolescents aged 13–18 years^(22)^. Positive associations have also been reported between breakfast skipping and vitamin and mineral intakes among Japanese adolescents^(23)^. However, in this paper, the relationship between breakfast skipping and diet by sex was not studied because male and female participants were analysed as a single group. Additionally, the intakes were not compared with the Japanese dietary reference intake (DRI) values. It has been reported that growth in adolescence differs by sex^(24)^, and that the influence of breakfast skipping on diet is different between adolescent boys and girls (Belgian study)^(22)^. In girls, a focus on body image is thought to lead to breakfast skipping^(25)^. Therefore, it is necessary to examine the influence of breakfast skipping on nutrient intake adequacy by sex, and girls need special attention in this regard. Furthermore, previous reports have been limited to the relationship between breakfast skipping and nutrient intake of a few select nutrients or of nutrients in general; there are no reports on the association between breakfast skipping and the overall adequacy of habitual nutrient intake. Therefore, we aimed to examine the relationship between breakfast skipping and adequacy of total habitual nutrient intake among junior high school female students.

## Methods

### Study design and participants

A set of two self-administered questionnaires (i.e. a diet history questionnaire and lifestyle questionnaire) were distributed by teachers to 742 junior high school female students belonging to three schools in Kanto, Japan in June 2016. Students were asked to answer the questionnaires on their own, or in cooperation with their parents, if necessary. The completed questionnaires were examined by the research staff, and those with missing information were returned to the students for completion. Both questionnaires were completed by 575 female students.

We excluded students with missing data (*n* 17), with milk allergies (*n* 8), and those with a reported energy intake less than half the energy requirement for the lowest physical activity category, according to the Japanese DRI or that equal to or more than 1·5 times the energy requirement for the highest physical activity category (<4498 kJ/d or ≥16 945 kJ/d; *n* 34)^(26)^. Thus, the final participants consisted of 516 junior high school female students. Written informed consent was obtained from all participants and their parents. This study was conducted according to the guidelines laid down in the Declaration of Helsinki and all procedures involving human subjects were approved by the Ethics Committee of Seitoku University, Japan (approval no. H27U056).

### Frequency of meals and snacks

Participants reported the frequency of breakfast eating per week in the lifestyle questionnaire, by answering the question: ‘How many times per week do you have breakfast?’. The definition of breakfast was the first meal of the day, eaten before or at the start of daily activities (e.g. errands, travel and work), within 2 h of waking, typically no later than 10.00 hours, and of an energy level between 20 and 35 % of total daily energy needs, which has been defined by Timlin & Pereira^(27)^. In the present study, we classified participants into two groups according to the frequency of breakfast eating: breakfast consumers group (seven times/week) and breakfast skippers group (0–6 times/week).

Using the lifestyle questionnaire, participants were also queried about the weekly frequency of lunch, dinner and snack consumption before and after dinner by means of the question ‘How many times per week do you have lunch, dinner, snacks before dinner and snacks after dinner?’. Due to the lack of any standard definition of lunch and dinner time in the literature, we defined lunch and dinner as meals eaten from 12.00 to 14.00 hours and from 16.00 to 20.00 hours, respectively.

### Dietary assessment

Habitual dietary intake during the preceding month was assessed using a brief self-administered diet history questionnaire for Japanese children and adolescents (BDHQ15y)^(28)^. The BDHQ15y was developed based on the adult version of the validated brief self-administered diet history questionnaire for Japanese adults (BDHQ) that enquires about dietary history during the preceding month^(29,30)^. The BDHQ15y is a four-page structured questionnaire comprising sixty-seven questions on the frequency of intake of food items commonly cooked and consumed in Japan. Daily food, energy and selected nutrient intakes were calculated using an *ad hoc* computer algorithm for the BDHQ15y based on the Standard Tables of Food Composition in Japan^(31)^. Validity of the BDHQ15y was verified by a study on the relationship between selected food intake and blood biomarker levels^(28)^. Food groups were categorised as per previous studies^(29,32)^.

Any self-estimated dietary assessment cannot avoid under- or over-reporting of dietary intake^(33,34)^. Therefore, we adjusted the reported dietary intakes based on the assumption that each participant reported the estimated energy requirement (EER) when the physical activity level was at the second level to render the comparison between the reported nutrient intake and the Japanese DRI values practically possible. The following formula was used: dietary intake (unit/d) = reported dietary intake (unit/d)/reported energy intake (kJ/d) × EER (kJ/d). The percentage of daily energy intake was calculated using the crude value for total fat and carbohydrate intake. Additionally, food intake values were energy-adjusted using the density method (i.e. the percentage of energy for energy-providing nutrients and their amounts per 4184 kJ for food groups and other nutrients) to minimise the influence of dietary misreporting.

### Determination of habitual nutrient intake inadequacy

Inadequacy of intake for each nutrient was determined by comparing nutrient levels with each dietary reference value according to the Japanese DRI using a previously reported method^(35–37)^. Different types of dietary reference values comprising the Japanese DRI were established according to their purpose. The estimated average requirement (EAR) is set to avoid insufficient intake of nutrients, whereas the tentative dietary goal to prevent lifestyle-related diseases (DG) is set to prevent non-communicable diseases.

An intake level below the EAR was considered as inadequate, and a cut-point method was used for the following fourteen nutrients with known EAR: protein, vitamin A expressed as retinol activity equivalents, vitamin B_1_, vitamin B_2_, niacin expressed as niacin equivalents, vitamin B_6_, vitamin B_12_, folate, vitamin C, Ca, Mg, Fe (EAR reference values for during menstruation because 87·7 % female adolescents aged 12–15 years have experienced their first menstruation^(38)^), Zn and Cu. For the following five nutrients, the intake level outside the range of DG values was considered as not meeting the standard DG: total fat, carbohydrate, total dietary fibre, Na expressed as salt-equivalents, and K.

### Other variables

Using the BDHQ15y, participants were asked about their body weight and height. The BMI was calculated as weight (kg) divided by the square of height (m). In the BDHQ15y, participants also reported the weekly frequency of performing exercises such as sports club activities for the past month (every day, 4–6 d/week, 2–3 d/week, 1 d/week, or never). Additionally, participants reported on the following variables by completing a self-administered lifestyle questionnaire: employment status of father (full-time, part-time, housekeeping, or other), and employment status of mother (full-time, part-time, housekeeping, or other).

### Statistical analysis

All statistical analyses were performed using the IBM SPSS statistics software package (version 22.0; SPSS Inc.). All reported *P* values were two-tailed, and a *P* value of 0·05 was considered as statistically significant. The differences in characteristics between breakfast consumers and breakfast skippers were compared using the χ^2^ test for categorical variables and the independent *t* test for continuous variables. Potential confounding factors considered in the adjusted model were school type (public or private), frequency of lunch skipping (0 d/week or 1–7 d/week), dinner skipping (0 d/week or 1–7 d/week), and snack eating before dinner (0 d/week or 1–7 d/week) which were found to be substantially different (*P* < 0·05) between groups categorised by the frequency of breakfast consumption, and snack eating after dinner (0 d/week or 1–7 d/week) observed in associations with nutrient or food intake. The difference in the mean nutrient intake between the two groups was compared using an independent *t* test in the crude model, while a covariate analysis (ANCOVA) was performed to adjust for confounding variables (such as school type, frequency of lunch skipping, dinner skipping, snack eating before dinner, and snack eating after dinner) in the adjusted model. The nutritional inadequacy of each nutrient intake was represented as the proportion of participants whose intake was below the EAR or outside the range of the DG in each group. The χ^2^ test for the crude model and the logistic regression analysis adjusted for school type, frequency of lunch skipping, dinner skipping, snack eating before dinner, and snack eating after dinner for the adjusted model were used to examine the difference in the prevalence of not meeting DRI between breakfast consumers and skippers.

The overall nutritional inadequacy of participants was determined based on the number of nutrients that did not meet the DRI values of the fourteen nutrients with EAR and five nutrients with DG. Differences in the number of nutrients that did not meet DRI between the breakfast consumers and skippers groups were assessed by the independent *t* test in the crude model and a covariate analysis to adjust for school type, frequency of lunch skipping, dinner skipping, snack eating before dinner, and snack eating after dinner in the adjusted model.

## Results

The basic characteristics of the participants are shown in Table 1. The incidence of breakfast skipping was significantly lower in students attending public junior high school than in those attending private junior high school (44·9 *v.* 55·1 %; *P* = 0·002). Students having breakfast every day were likely to have lunch and dinner every day in comparison with those who did not necessarily eat breakfast every day (both *P* < 0·001). Additionally, the proportion of students eating snacks before dinner every day was significantly higher in breakfast consumers than in breakfast skippers (*P* = 0·003). The proportion of students skipping breakfast 1–2 d/week was approximately 60 % among breakfast skippers. There were no significant differences in grade, BMI, the number of participants who engaged in habitual exercise, energy intake, working status of the father and mother, and the proportion of eating snacks after dinner every day, between breakfast consumers and skippers.

**Table 1.** Characteristics of study participants categorised into breakfast consumers and skippers (*n* 516) (Numbers and percentages; mean values and standard deviations)

	Breakfast consumers (*n* 427)		Breakfast skippers (*n* 89)		
	*n*	%	*n*	%	P*
Grade					0·688
1	149	34·9	27	30·3	
2	152	35·6	36	40·4	
3	126	29·5	27	30·3	
School type					0·002
Public	266	62·3	40	44·9	
Private	161	37·7	49	55·1	
Body height (cm)					0·389
Mean	154·4		154·6		
sd	8·1		6·3		
Body weight (kg)					0·637
Mean	45·5		46·5		
sd	7·6		7·3		
BMI (kg/m^2^)					0·909
Mean	19·3		19·4		
sd	6·5		2·6		
Energy intake (kJ/d)					0·176
Mean	8883		8410		
sd	2213		2598		
Number of days exercising					0·158
Every day	202	47·3	29	32·6	
4–6 d/week	61	14·3	17	19·1	
2–3 d/week	49	11·5	12	13·5	
1 d/week	39	9·1	10	11·2	
Never	76	17·8	21	23·6	
Work status of father					0·205
Full-time	372	87·1	73	82·0	
Others	55	12·9	16	18·0	
Work status of mother					0·821
Full-time	88	20·6	21	23·6	
Part-time	170	39·8	34	38·2	
Others	169	39·6	34	38·2	
Eating lunch every day					<0·001
Yes	417	97·7	69	77·5	
No	10	2·3	20	22·5	
Eating dinner every day					<0·001
Yes	414	97·0	71	79·8	
No	13	3·0	18	20·2	
Eating snacks before dinner every day					0·003
Yes	110	25·8	10	11·2	
No	317	74·2	79	88·8	
Eating snacks after dinner every day					0·278
Yes	29	6·8	9	10·1	
No	398	93·2	80	89·9	
Number of days skipping breakfast					–
1–2 d/week	–	–	53	59·6	
3–4 d/week	–	–	12	13·5	
5–6 d/week	–	–	11	12·4	
7 d/week	–	–	13	14·6	

* The *P* values are shown for the χ^2^ test for categorical variables and for the independent *t* test for continuous variables and pertain to comparisons between breakfast consumers and skippers.

The daily nutrient intake and nutrient intake inadequacy among 516 junior high school female students are shown in Table 2. In the crude model, most nutrient intakes except those of fat, carbohydrate, total dietary fibre, vitamin A, niacin, vitamin B_12_ and Na were significantly higher in breakfast consumers than in breakfast skippers. In the adjusted model, the carbohydrate intake of breakfast skippers was higher than that of breakfast consumers. The proportion of students having inadequacy of vitamin B_1_, vitamin B_2_, vitamin C, Ca, Zn and K was significantly lower in breakfast consumers than in breakfast skippers in the crude model, while the significant difference in vitamin A and Fe intake inadequacy was additionally found in the adjusted model. With reference to Na intake inadequacy alone, the proportion of participants showing inadequacy was higher in breakfast consumers group compared with that in the breakfast skippers group.

**Table 2.** Daily nutrient intakes and prevalence of not meeting estimated average requirement (EAR) and tentative dietary goal to prevent lifestyle-related disease (DG) among 516 female junior high school students categorised into breakfast consumers and skippers* (Mean values and standard deviations)

	Reference value†	Breakfast consumers (*n* 427)		Inadequacy‡ (%)	Breakfast skippers (*n* 89)		Inadequacy‡ (%)	Crude model		Adjusted model		
		Mean	sd		Mean	sd		*P*§	*P*‖	*P*¶	OR**	95 % CI
Nutrients with EAR												
Protein (g)	≥45	81	14	0	77	13	0	0·004	–	<0·001	–	–
Vitamin A (μg RAE)††	≥500	815	401	16·4	755	365	24·7	0·194	0·062	0·184	0·546	0·301, 0·989
Vitamin B_1_ (mg)	≥1·1	0·96	0·17	79·6	0·91	0·16	88·8	0·007	0·045	0·005	0·379	0·173, 0·831
Vitamin B_2_ (mg)	≥1·2	1·8	0·41	6·6	1·6	0·44	15·7	0·001	0·004	< 0·001	0·377	0·179, 0·794
Niacin (mg NE)‡‡	≥12	17	4·3	10·8	16	3·8	16·9	0·097	0·106	0·066	0·508	0·252, 1·022
Vitamin B_6_ (mg)	≥1·1	1·4	0·33	18·7	1·3	0·26	27·0	0·005	0·078	0·002	0·582	0·326, 1·038
Vitamin B_12_ (mg)	≥1·9	8·6	4·0	0·2	7·9	3·3	0	0·135	–	0·066	–	–
Folate (μg)	≥190	404	139	2·8	354	117	5·6	0·002	0·117	0·001	0·522	0·154, 1·771
Vitamin C (mg)	≥80	138	57	12·2	117	47	23·6	0·001	0·005	<0·001	0·479	0·256, 0·894
Ca (mg)	≥700	875	281	29·3	768	292	50·6	0·001	<0·001	<0·001	0·314	0·186, 0·530
Mg (mg)	≥240	290	58	19·7	271	46	21·3	0·004	0·719	0·001	0·777	0·428, 1·429
Fe (mg)	≥10·0	8·9	2·1	73·3	8·4	1·9	83·1	0·036	0·051	0·020	0·477	0·250, 0·912
Zn (mg)	≥7·0	10·0	1·4	0·9	9·4	1·4	4·5	0·001	0·013	<0·001	0·195	0·040, 0·952
Cu (mg)	≥0·6	1·3	0·21	0	1·2	0·20	0	0·049	–	0·041	–	–
Nutrients with DG												
Fat (% energy)	20–30	30	5·3	56·9	29	5·8	50·6	0·111	0·273	0·119	1·402	0·852, 2·308
Carbohydrate (% energy)	50–65	53	6·6	32·3	55	6·8	33·7	0·064	0·799	0·044	1·131	0·662, 1·932
Total dietary fibre (g)	≥16	13	3·9	80·6	12	3·2	84·3	0·098	0·415	0·060	0·692	0·355, 1·348
Na (salt-equivalent) (g)	<7·0	12·6	2·8	99·5	12·1	2·4	96·6	0·090	0·011	0·331	21·177	2·011, 222·995
K (mg)	≥2400	2851	707	29·0	2562	606	40·4	<0·001	0·034	<0·001	0·498	0·296, 0·839

RAE, retinol activity equivalents; NE, niacin equivalents; DRI, dietary reference intake.

* Adjustment of reporting error was performed according to the following: nutrient intake = reported nutrient intake/reported energy intake × estimated energy requirement.

† DRI for 12- to 14-year-old Japanese girls. The estimated energy requirement of physical activity level II is 10 042 kJ/d.

‡ Percentage of participants whose nutrient intake did not meet DG or EAR of DRI. Each nutrient intake was compared with the appropriate DRI value using the cut-point method.

§ *P* values are shown for independent *t* test to analyse differences of nutrient intake between breakfast consumers and skippers.

ǁ *P* values are shown for the χ^2^ test to analyse differences in the prevalence of participants with inadequate nutrient intake between breakfast consumers and skippers.

¶ *P* values are shown for covariate analysis to analyse difference of nutrient intake between breakfast consumers and skippers adjusted for confounding variables of school type (public or private), frequency of lunch skipping (0 d/week or 1–7 d/week), dinner skipping (0 d/week or 1–7 d/week), and snack eating before dinner (0 d/week or 1–7 d/week).

** Multivariate adjusted OR about nutrient intake inadequacy of breakfast skipper with reference to breakfast consumers were calculated by adjusting for frequency of school type (public or private), lunch skipping (0 d/week or 1–7 d/week), dinner skipping (0 d/week or 1–7 d/week), snack eating before dinner (0 d/week or 1–7 d/week) and snack eating after dinner (0 d/week or 1–7 d/week).

†† Sum of retinol, β-carotene/12, α-carotene/24, and cryptoxanthin/24.

‡‡ Sum of niacin and protein/6000.

Table 3 shows the overall nutritional inadequacy among female junior high school students in the breakfast consumers and skippers groups. The total number of nutrients not meeting EAR among breakfast consumers was lower than that among breakfast skippers in the adjusted model, while the total number of nutrients not meeting DG did not differ significantly between the two groups.

**Table 3.** Number of nutrients not meeting tentative dietary goal (DG) and estimated average requirement (EAR) among 516 female junior high school students categorised into breakfast consumers and skippers (Mean values and standard deviations)

	Breakfast consumers (*n* 427)		Breakfast skippers (*n* 89)		Crude model: P*	Adjusted model: *P*†
	Mean	sd	Mean	sd		
Total number of nutrients not meeting EAR	2·7	2·1	3·6	2·4	0·074	<0·001
Total number of nutrients not meeting DG	3·0	1·0	3·1	1·1	0·260	0·632

* *P* values are shown for the independent *t* test to analyse differences between breakfast consumers and skippers.

† *P* values are shown for covariate analysis to analyse differences between breakfast consumers and skippers adjusted for confounding variables of school type (public or private), frequency of lunch skipping (0 d/week or 1–7 d/week), dinner skipping (0 d/week or 1–7 d/week), snack eating before dinner (0 d/week or 1–7 d/week) and snack eating after dinner (0 d/week or 1–7 d/week).

The habitual food groups intake among female junior high school students is shown in Table 4. In the crude model, the daily intakes of total vegetables, green and yellow vegetables, other vegetables, green tea and dairy products were significantly higher among breakfast consumers than among breakfast skippers, while noodle and soft drink intakes were significantly lower among breakfast consumers than among breakfast skippers. In addition to the above-mentioned differences found with the crude model, higher fruit intake among breakfast consumers was found with the use of the adjusted model.

**Table 4.** Daily food group intakes among 516 female junior high school categorised into breakfast consumers and skippers (g/4184 kJ)* (Mean values and standard deviations)

	Breakfast consumers (*n* 427)		Breakfast skippers (*n* 89)		Crude model: *P*†	Adjusted model: *P*‡
	Mean	sd	Mean	sd		
Cereals	206·8	62·6	218·3	69·9	0·123	0·071
Rice	159·2	67·4	159·5	71·2	0·973	0·657
Bread	21·9	13·4	18·9	13·0	0·056	0·101
Noodles	25·7	16·9	39·9	34·7	<0·001	<0·001
Pulses	31·0	19·8	28·3	17·2	0·224	0·201
Potatoes	15·2	10·5	15·3	10·6	0·945	0·517
Sugar	2·5	2·3	2·5	2·4	0·763	0·404
Confectionery	43·5	28·3	46·1	28·4	0·429	0·366
Fat and oil	7·8	3·3	7·8	2·9	0·874	0·923
Fat	0·6	0·9	0·6	1·1	0·646	0·465
Oil	7·1	3·1	7·3	2·8	0·753	0·741
Fruits	35·6	33·2	28·6	25·0	0·061	0·028
Total vegetables	129·5	76·0	110·2	58·6	0·025	0·011
Green and yellow vegetables	48·5	32·6	40·0	24·6	0·022	0·017
Other vegetables	65·6	40·8	54·9	34·3	0·022	0·007
Pickled vegetables	4·9	6·1	5·7	6·2	0·233	0·292
Mushrooms	4·7	5·0	4·4	3·8	0·499	0·329
Seaweed	5·8	5·5	5·2	4·9	0·308	0·129
Beverages	321·9	175·6	321·0	195·8	0·964	0·606
Fruit and vegetable juice	31·2	50·3	23·7	34·6	0·185	0·129
Green tea	205·8	125·9	168·8	133·1	0·013	0·017
Black tea	37·9	80·7	48·4	94·2	0·280	0·831
Soft drinks	47·0	68·9	80·1	107·3	<0·001	0·001
Fish and shellfish	31·2	17·7	29·4	16·6	0·370	0·264
Meat	38·6	17·5	37·5	17·0	0·592	0·473
Eggs	18·4	10·3	17·5	11·3	0·464	0·454
Dairy products	131·6	94·8	104·2	103·7	0·015	0·004

* Adjustment of reporting error was performed according to the following: food group intake = reported food group intake/reported energy intake × 4184 (kJ).

† *P* values are shown for the independent *t* test to analyse differences between breakfast consumers and skippers.

‡ *P* values are shown for covariate analysis to analyse differences between breakfast consumers and skippers adjusted for confounding variables of school type (public or private), frequency of lunch skipping (0 d/week or 1–7 d/week), dinner skipping (0 d/week or 1–7 d/week), eating a snack before dinner (0 d/week or 1–7 d/week) and snack eating after dinner (0 d/week or 1–7 d/week).

## Discussion

The present study examined the association of breakfast skipping with nutrient adequacy in female junior high school students. We found that junior high school female students who ate breakfast every day had higher intakes of most nutrients and lower inadequacy of nutrient intakes than those who skipped breakfast. To the best of our knowledge, the present study is the first to examine the relationship between breakfast skipping and overall nutrient intake adequacy among female junior high school students in Asia including Japan.

In the present study, the rate of breakfast skipping among female students was 17·2 %; this result is in agreement with those published from other countries including the USA (18·0 %)^(39)^, UK (19·9 %)^(21)^ and Belgium (16·9 %)^(22)^. However, the rate of breakfast skipping in the present study was inconsistent with the rate previously reported for Japanese adolescents (2·3 %)^(23)^. The possible reasons for this could be differences in participant characteristics and in the definition of breakfast skipping between studies. The participants in the previous study included both girls and boys^(23)^. Another previous study has reported that the proportion of breakfast skippers among adolescents was higher in girls than in boys^(21,22)^. Thus, the higher breakfast skipping rate observed in the present study may have been because this study only included girls. Additionally, breakfast skippers in the previous study were defined as individuals who reported that they did not consume any foods or beverages at breakfast in the 1-d dietary records^(23)^; however, in this study, individuals whose frequency of breakfast consumption was 0–6 times/week were defined as breakfast skippers. The difference in the definition of breakfast skipping between studies may have produced the differences in results.

The rate of breakfast skipping was significantly higher among students from private schools than among those from public schools. It has been reported that the proportion of private/public junior high school students is directly proportional to the commute time^(40,41)^. Additionally, one of the reasons for breakfast skipping was lack of time (for eating breakfast) in the morning^(17)^. This may explain the difference in the rate of breakfast skipping between public and private school students; however, further studies are needed to examine the influence of the school type on breakfast skipping. It was observed that the rate of lunch or dinner skipping was higher among breakfast skippers than among breakfast consumers in the present study. A study of Japanese adults reported that breakfast skippers were likely to have the unhealthy lifestyle habit such as irregular time of eating dinner^(42)^. This result was consistent with that of the present study. However, the proportion of students eating snacks before dinner every day was higher in the breakfast consumers than in the skippers group, which was not similar to the finding from the previous study which reported a lower proportion of people having snacks every day among breakfast consumers among Japanese adults^(42)^. The difference in results between the two studies might be due to the fact that the energy requirement was different for adults and junior high school students^(43)^. However, the content of the snack was not explained, and, thus, further studies are needed.

A Japanese study has reported that the intakes of vitamins and minerals were higher among breakfast consumers than among breakfast skippers^(23)^. Additionally, a UK study reported that the intakes of folate, vitamin C, Ca and Fe were higher in breakfast consumers than in breakfast skippers^(21)^. These results are consistent with the results of the present study. In the present study, it was observed that the intakes of most nutrients excluding fat, carbohydrate, total dietary fibre, vitamin A, niacin, vitamin B_12_ and Na were higher among breakfast consumers than among skippers in crude model analysis. In the present study, habitual vitamin A, niacin, vitamin B_12_, fat, total dietary fibre and Na intake were the only six parameters which were not significantly different between the two groups both in the crude and the adjusted model. Regarding vitamin A intake, one reason for the lack of significant difference between groups may be that the intake was highly varied between individuals and well as in a single individual over time^(44)^. Thus, it is possible that the results were influenced by characteristics of food intake pattern, and not necessarily by the meal itself. Therefore, further study will be needed to reveal the food sources and intake levels as per each of the food sources for vitamin A among Japanese adolescents. However, in the adjusted model, the proportion of participants in our study who showed vitamin A intake adequacy was higher in breakfast consumers than in breakfast skippers. This result was similar to that from a previous study which suggested a difference of vitamin A intake adequacy based on breakfast consumption, among Canadian girls aged 4–18 years^(45)^. Therefore, evidence suggests that vitamin A nutrient inadequacy is affected by the frequency of breakfast consumption.

On the one hand, no differences were observed in the habitual niacin, vitamin B_12_, fat and total dietary fibre intake adequacy. The results of niacin and vitamin B_12_ intakes in the present study were consistent with those of a previous study which observed no differences in niacin and vitamin B_12_ intakes between breakfast consumers and skippers among adolescents^(23)^. Regarding fat intake, the result of no difference between breakfast consumers and skippers in the present study is consistent with a UK adolescents study^(21)^ and Japanese study^(23)^ which assessed fat as macronutrient balance (% energy). Thus, the fat intakes in terms of macronutrient balance (% energy) may not be associated with breakfast skipping among Japanese female adolescences. Additionally, for total dietary fibre, the proportion of people who meet total dietary fibre value of DRI was low. This may partly explain the deficiency in total dietary fibre which was reported among many Japanese adolescent girls and young women^(36,46)^. Therefore, total dietary fibre intake may be deficient regardless of breakfast consumption, and breakfast skipping is unlikely to be the most significant factor to influence this outcome. However, Na inadequacy was higher among breakfast consumers than among breakfast skippers. In the present study, breakfast consumers had a higher vegetable intake than did breakfast skippers. Vegetables are cooked with salt seasoning like miso soup served with rice in the traditional Japanese dietary pattern^(47)^, which was reported as the main source of Na in the Japanese diet^(48)^. This may partly explain the present result that the inadequacy of Na intake may be higher among breakfast consumers.

A higher intake of carbohydrates was observed among breakfast skippers than in consumers in adjusted model analysis. This may be partly explained by the higher intakes of noodles and soft drinks among breakfast skippers in the present study. This result was consistent with a previous study which reported that young Japanese women aged 18–20 years who sleep late are more likely to skip breakfast and tend to eat noodles^(49)^. Moreover, sleep restriction has been reported to be associated with a carbohydrate intake preference^(50)^. Breakfast skippers may have a short sleep time^(49)^, which may have increased carbohydrate intake. Further study is needed to explain the higher carbohydrate intake among breakfast skippers in consideration of bedtime and wake-up time.

There was little to no nutrient inadequacy of protein, vitamin B_12_ and Cu among the entire study population (incidence: 0 %). The proportion of Japanese adolescent girls^(46)^ and young women^(36)^ with insufficient intakes of these nutrients was reported to be very small, and thus these reports are consistent with our present findings. In other words, protein, vitamin B_12_ and Cu might be nutrients which are sufficiently consumed by Japanese people, and the adequacy of these nutrients may not be related to breakfast skipping.

The habitual intakes of vitamin B_6_, folate and Mg were higher among breakfast consumers than among breakfast skippers, which was consistent with the result that the contribution of vitamin B_6_, folate and Mg intakes at breakfast was reported to be approximately 20 % of total intake of these nutrients^(39)^. However, the nutrient intake adequacy of vitamin B_6_, folate and Mg intakes was not different between the two groups both in crude model and adjusted model analyses. A previous study in Japanese adolescents reported that there only a few people who did not meet the EAR of vitamin B_6_, folate and Mg^(46)^. The nutrient intakes in the previous study were assessed by dietary records, while our study used a diet history questionnaire. It is possible that this difference in dietary survey methods may have affected the results. Additionally, the nutrient adequacy of vitamin B_6_, folate and Mg differs considerably based on dietary patterns among Japanese young women^(35)^. Therefore, further study will be needed to reveal the food sources for vitamin B_6_, folate and Mg among Japanese adolescents; additionally, the influence of type of dietary pattern on intake levels of those nutrients among adolescent girls also needs to be evaluated.

Regarding vitamin B_1_, vitamin B_2_, vitamin C, Ca, Fe, Zn and K, both the habitual nutrient intakes and nutrient adequacy were observed to be different between breakfast consumers and skippers. These results were consistent with those from a previous study^(21–23,44)^. Therefore, the intakes and deficiency of many vitamins and minerals may be affected by breakfast skipping.

The overall nutritional inadequacy was observed in the difference of the total number of nutrients not meeting EAR between breakfast consumers and skippers, but not those not meeting DG. A difference in energy intake between breakfast consumers and skippers was not observed. About half of the nutrients for which DG is set are energy-providing nutrients such as carbohydrate and fat. On the other hand, most nutrients for which EAR is set are vitamins and minerals. For a majority of these, there was observed a difference of nutritional inadequacy for each nutrient between breakfast consumers and skippers in the present study. Additionally, these results that were observed in the difference of vitamins and minerals intake adequacy were similar to the previous study results that showed the difference of vitamins and minerals intake adequacy such as vitamin C, Ca and Fe with or without breakfast^(21)^. These may partly explain the present result of overall nutritional inadequacy.

Higher intakes of fruit, vegetables, green tea and dairy products and lower intakes of noodles and soft drinks were observed among breakfast consumers than among breakfast skippers. The results of the present study were consistent with those from previous studies which reported higher intakes of fruits, vegetables and dairy products^(23,39,51)^, and lower intakes of soft drinks^(23)^, among breakfast consumers. It has also been reported that Japanese tend to consume green tea during meals^(52)^. However, breakfast skippers have been reported to be higher consumers of soft drinks^(53)^. These may partly explain the present study results that there was no difference of beverage consumption between breakfast consumers and skippers, while breakfast consumers had higher green tea consumption and lower soft drink consumption than breakfast skippers in the present study. Moreover, a dietary pattern including higher vegetable intake was reported to be a healthy dietary pattern, while that consisting of higher noodle intake was reported to be an unhealthy dietary pattern^(54)^. Consequently, the consumption of breakfast every day may lead to a healthier dietary pattern among female junior high school students.

Several limitations of this study need to be mentioned. First, the participants in the study were not sampled randomly from the general population, and the survey area was restricted to a single region in Japan. The participants were therefore probably not representative of Japanese adolescents. Second, we used the BDHQ15y to assess dietary intake, although its ability to estimate dietary intakes has been validated on a limited number of foods and nutrients. Third, we could not include intake from dietary supplements in the analysis because reliable composition tables of dietary supplements were lacking in Japan. Fourth, household income, education, sleep duration, wake-up time, bedtime and nutritional habits of participants' parents were not investigated. These factors have been reported to influence children's diets^(49,55–57)^. Although the employment status of participants’ parents was examined in this study, this information is not necessarily equivalent to data on household income. Finally, we did not examine whether the participants had experienced their first menstruation, although the EAR for Fe changes depending on menstruation. A previous study reported that 585 out of 4769 female students (12·3 %) aged 12–15 years had not experienced their first menstruation, and the majority of these girls were 12 years old^(38)^. Because junior high school female students are likely to have their first menstruation shortly, they would need higher Fe intake. Thus, we considered that the EAR for Fe for menstruating women was a more appropriate reference value to assess nutritional adequacy in this study.

### Conclusions

This cross-sectional study showed that breakfast skipping was related to the deficiency of many vitamins and minerals such as that of vitamin A, vitamin B_1_, vitamin B_2_, vitamin C, Ca, Fe, Zn and K among Japanese female junior high school students. Additionally, breakfast consumption was likely to lead to a healthier dietary pattern. Therefore, further studies are needed to clarify the factors related to skipping breakfast to devise a strategy for improving dietary behaviour among adolescent girls.
